# Nanocomposite Hydrogel with Tantalum Microparticles for Rapid Endovascular Hemostasis

**DOI:** 10.1002/advs.202003327

**Published:** 2020-11-30

**Authors:** Hassan Albadawi, Izzet Altun, Jingjie Hu, Zefu Zhang, Anshuman Panda, Han‐Jun Kim, Ali Khademhosseini, Rahmi Oklu

**Affiliations:** ^1^ Minimally Invasive Therapeutics Laboratory Department of Vascular and Interventional Radiology Mayo Clinic Phoenix AZ 85054 USA; ^2^ Terasaki Institute for Biomedical Innovation Los Angeles CA 90024 USA

**Keywords:** angiography, catheter, embolization, hemorrhage, intervention

## Abstract

Endovascular embolization to treat vascular hemorrhage involves pushing coil‐shaped metal wires into the artery repeatedly until they are densely packed to slow the blood flow and clot. However, coil embolization is associated with high rebleeding rates, unpredictable economics and, most importantly, they rely on the patient's ability to make a clot. These issues are exacerbated when the patient is anticoagulated or coagulopathic. A novel bioengineered tantalum‐loaded nanocomposite hydrogel for gel embolic material (Ta‐GEM) that can be rapidly delivered using clinical catheters for instant hemostasis regardless of the coagulopathic state is reported. Ta‐GEM formulation is visible by most of the clinically available imaging modalities including ultrasound, computed tomography, magnetic resonance imaging, and fluoroscopy without significant artifact. In addition, Ta‐GEM can be retrieved, allowing temporary vascular occlusion, and it can be used to rescue cases of failed coil embolization. Ta‐GEM occlusion of first‐order arteries such as the renal artery and iliac artery in a swine model is found to be safe and durable; by 28 days, 75% of the injected Ta‐GEM in the arterial lumen is replaced by dense connective tissue. Altogether, this study demonstrates that Ta‐GEM has many advantages over the current technologies and has potential applications in clinical practice.

## Graphical abstract

Endovascular embolization is performed to treat various vascular diseases including hemorrhage, aneurysms, and vascular malformations. A tantalum microparticle‐loaded shear‐thinning gel embolic material (Ta‐GEM) that can be rapidly delivered into diseased arteries using clinical catheters to achieve rapid hemostasis is reported. In large animal models, Ta‐GEM leads to a durable occlusion without recanalization and outperforms coils and gelfoam under coagulopathic conditions.

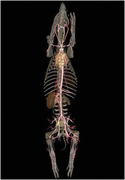

## 1 Introduction

Hemorrhage in a setting of anticoagulation (ACA) is a serious medical emergency associated with high morbidity and mortality. In the United States alone, there are over 2.6 million people with atrial fibrillation on ACA.^[^ ^1^ ^]^ More than 30 million prescriptions for coumadin are written annually, and prescriptions for newer oral anticoagulants are also increasing; the bleeding frequency for coumadin alone is estimated to be 15–20% per year.^[^ ^2^ ^]^ Bleeding risk is even higher in patients with mechanical valves and cardiac assist devices (CAD).^[^ ^3^ ^]^ In fact, up to 40% of patients with CAD develop gastrointestinal bleeding (GIB).^[^ ^4^ ^]^ The most common causes of GIB in these patients are inflammatory, vascular malformations, or trauma‐related etiologies.^[^ ^5^, ^6^ ^]^ Mortality from GIB drastically increases to fourfold if bleeding occurs during hospitalization for another serious disease and, if it is recurrent bleeding, mortality increases tenfold.^[^ ^6^ ^]^ Transcatheter arterial embolization has emerged as one of the safest and most efficient methods in treating hemorrhage. Embolization is the intentional endovascular occlusion of an artery or vein. The technique has been adopted for indications across a wide variety of conditions; examples of clinical applications include nontraumatic and traumatic hemorrhage, saccular aneurysms, vascular malformations, devascularization or ablation of benign and malignant tumors (e.g., hepatic tumors, uterine fibroids, prostate, etc.), and endoleak management.^[^ ^7^ ^]^

There are several types of embolic agents used today and these can be divided into solid and liquid embolic materials. The most common type of solid embolic material used to treat focal hemorrhage or saccular aneurysms are coils. Coils have a variety of shapes, lengths, and thickness; they also require specific catheters to deliver them and require special equipment to detach them into the vessel. Often multiple coils are required and, in some cases many dozens, adding significantly to healthcare costs. Coils, however, are associated with many drawbacks; rebleeding or break‐through bleeding rates can be as high as 47% contributing to increased morbidity and mortality especially in the coagulopathic patient.^[^ ^8^ ^]^ Coil migration and coil compaction can also occur, which can lead to rebleeding. In addition, they require highly skilled physicians to perform these cases, the deployment time of these coils is lengthy, exposing the patient and the staff to high radiation doses, and the coils are costly limiting access to such life‐saving technology to larger medical centers.^[^ ^9^ ^]^

Introduced 15 years ago, liquid embolics are specially formulated materials designed to self‐solidify upon deployment in situ. Once injected, liquid embolics undergo a transition to form a solid based on physicochemical mechanisms, including polymerization, precipitation, and crosslinking through ionic or thermal processes. The two frequently used liquid embolic agents include Trufill n‐BCA system (Johnson & Johnson) and Onyx (Medtronic). n‐BCA polymerizes into an adhesive, nonbiodegradable solid material via an anionic mechanism; Medtronic's nonadhesive liquid embolic, Onyx, consists of an ethylene vinyl alcohol copolymer dissolved in dimethyl sulfoxide. Unlike n‐BCA, which polymerizes almost immediately, Onyx has a slow in situ solidification rate that allows for more prolonged injection, enhancing distal penetration. In 2012, FDA issued a warning about the risk of catheter entrapment when using Onyx preventing the removal of the catheter inside the artery in the patient's brain.^[^ ^10^ ^]^ Liquid embolics can also have recanalization rates of up to 36%,^[^ ^11^ ^]^ they are associated with leakage during injection that can cause nontarget embolization, angiotoxicity, and the possibility of necrosis. The lack of a reliable tool for embolization necessitates a more effective technique that is occlusive regardless of coagulation capability, less time‐consuming to deliver, easier to use, safe, and cost‐effective.

To date, there is no retrievable liquid embolic material or coils following delivery and both liquid embolics and coils create significant artifacts when imaged by computed tomography (CT), magnetic resonance imaging (MRI), or ultrasound (US); this limits assessment of the intervention. Catheter‐based injectable hydrogels can serve as a promising alternative that overcome the limitations of embolics used in the clinic today. Hydrogels including those derived from alginate, chitosan, or decellularized tissue have been described previously.^[^ ^7^ ^]^ In line with these efforts, we recently reported a gelatin‐based nanocomposite hydrogel embolic agent which was primarily tested in the venous system of three pigs.^[^ ^12^ ^]^ However, these hydrogels have not been rigorously tested in the arterial system as an alternative to coil embolization.

The arterial system poses many challenges to achieve successful embolization to treat hemorrhage which may be secondary to pancreatitis, GI ulcer, a tumor, a vascular malformation, or injury resulting from penetrating or blunt trauma. Arteries have blood velocities and pressures that are significantly higher than the venous system, and arteries that may require embolization can vary in size ranging from sub‐millimeter in diameter to a centimeter. They may be tortuous and fragile, making them susceptible to complications during embolization arising from vasospasm, thrombosis, dissection, and rupture.^[^ ^7^ ^]^ Therefore, the main challenge to developing an effective arterial embolic agent is the versatility that the diseased or injured artery requires to achieve embolization.

The ideal embolic agent should have the capability to occlude wide range of arterial diameters without fragmentation or migration to avoid potential nontarget embolization, it should be retrievable to allow temporary embolization and rescue any accidental or off‐target injections, achieve intrinsic occlusion without relying on the body's ability to form a blood clot, and be visible on imaging modalities without artifact so that they can be followed over time to assess outcomes of the intervention. To date, there is no clinically used hydrogel‐based embolic agent; a major obstacle to using these hydrogels is achieving the capability to inject them through syringes connected to clinical catheters that can vary in diameters from several hundred micrometers to several millimeters at lengths exceeding 100 cm. To use hydrogels, it is important to optimize conditions that promote arterial occlusion without changing the physical properties of the biomaterial such as the injection force and its shear stress, while being detectable in multiple imaging modalities including CT, MRI, and US. Visibility of the hydrogel during intra‐arterial delivery is a critical property to prevent nontarget embolization that can lead to ischemia, tissue loss, and even death. In addition, when accidental delivery or nontarget embolization does occur, the ability to retrieve the hydrogel would be highly desirable.

Here, we aimed to develop a gel embolic material (GEM) comprised of nanosilicates, gelatin, and tantalum (Ta) microparticles that can be rapidly delivered via clinical catheters. Previously, we demonstrated that GEM has properties suitable for catheter‐based intravascular embolization, and exhibits high stability and excellent hemostatic properties.^[^ ^12^, ^13^ ^]^ In addition, the composition of gelatin and nanosilicate platelets was adjusted to evaluate the physical properties, biocompatibility, and biodegradability of GEMs that can be applied via various sizes of catheters.^[^ ^14^, ^15^ ^]^ Although our previous study showed mechanical and biological aspects of the GEM as a promising embolic agent, the study was mainly characterized in the venous system of animal models. In particular, considering that the microphysiological environment of the arterial system is markedly different from the venous system including the blood pressure, the absence of venous valves, and rates of blood flow, it was necessary to test the performance of the GEM in the arterial system. In addition, image guided visualization during delivery of GEM for clinical application was not optimized. To this end, Ta microparticles were loaded into GEM to develop advanced, clinically relevant embolic agent which can be detected in real time through multiple imaging devices with improved stabilization. The performance of Ta‐GEM was rigorously tested in vitro and in vivo in anticoagulated large animals and compared against coils. The versatility of Ta‐GEM to be retrievable as well as to embolize high velocity, high‐pressure arteries were also studied including its ability to rescue failed coil embolization. To demonstrate the durability of the occlusion and its biodegradability, Ta‐GEM implanted in porcine arteries were monitored for 28 days and imaged by CT imaging. Also, to determine whether Ta‐GEM recanalizes over time, embolization of an end‐organ artery such as the renal artery was performed and analyzed using CT volumetric studies, micro‐CT imaging, and histology. These results were compared to those of clinically approved gelfoam embolic agents.

## 2 Results

### 2.1 Development of Ta‐GEM for Enhanced Visibility

Clinically, metallic coils used today for embolization are visible during fluoroscopy; however, they produce significant artifacts with CT imaging, MRI, and US. The inability to assess the outcome of the intervention following embolization due to artifact is a major setback in clinical practice today. Therefore, we tested a formulation of Ta‐GEM that would allow material and local anatomical visualization in all imaging modalities, including CT, MRI, US, and fluoroscopy without significant artifact. In our previous experiments, GEM made with 10% iohexol was inadequate for visualization with fluoroscopy imaging. To improve visualization of GEM, we focused on Ta particulates because it is already in clinical use with various medical devices.^[^ ^16^, ^17^ ^]^ Ta has been used as an alternative contrast agent to iodinated agents because it is biologically safe and excreted in urine.^[^ ^18^ ^]^ Since Ta can be used in the form of microparticles^[^ ^19^ ^]^ and nanoparticles,^[^ ^16^, ^18^ ^]^ the level of visualization of Ta‐GEM was evaluated and compared to the iohexol‐loaded GEM.

**Figure 1:**
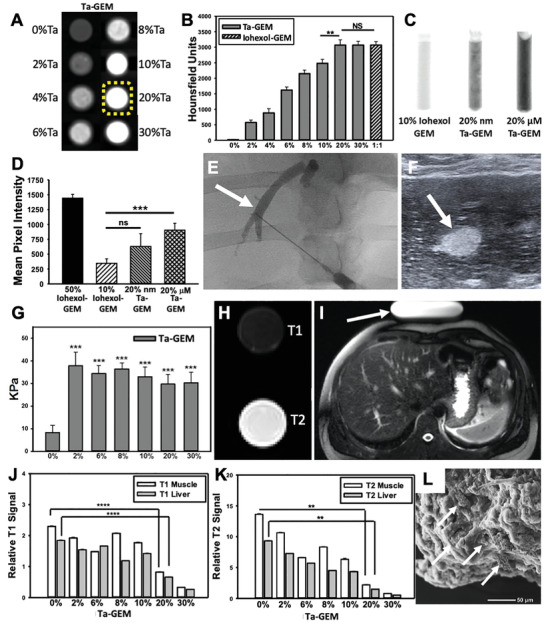
Assessing GEM visibility with multiple imaging modalities. A) CT axial images of eight syringes loaded with 0–30%Ta‐GEM inside a phantom simulating the human chest are shown; B) 20%Ta GEM (yellow dashed outline) demonstrates similar Hounsfield units when compared to 30% Ta‐GEM or 50% iohexol‐GEM (*n* = 4). C,D) Fluoroscopic images of syringes loaded with GEM containing 10% iohexol, 20% Ta nanoparticles (Nano‐Ta), or 20% Ta microparticles (Micro‐Ta); D) quantitation of the mean pixel intensity on fluoroscopy images show 20% micro‐Ta produce sufficient intensity (*n* = 4); 50% iohexol is included as a comparison because it is a concentration used clinically. E,F) Ultrasound and fluoroscopic images of the same area (arrow) during percutaneous hepatic vein embolization in a pig using 20% Ta‐GEM. G) Graphic summary of shear wave elastography of GEM containing 0–30% Ta microparticles (*n* = 3). H) Transverse view of standard T1 and T2 MRI of syringe filled with 20% Ta‐GEM showing attenuation for T1‐sequence and bright signal for T2‐sequence. I) T1 and T2 signal intensity of muscle and liver was calculated relative to an MRI phantom (arrow). J,K) The T1 and T2 signal of 20% Ta GEM relative to the signal calculated in (I) is depicted in the graphs; these indicate that the signal intensity of Ta‐GEM is greater than muscle or liver suggesting that it will be visible by MRI. L) Scanning electron microscopy of 20%Ta‐GEM (white arrows, Ta microparticles). Data are mean ± SD (**p < 0.01, ***p < 0.001, ****p < 0.0001).

Ta microparticles with an average size of 2 µm or Ta nanoparticles that are <25 nanometer in size were mixed with GEM to form Ta‐GEM (e.g., 2–30% w/w Ta‐GEM) using a SpeedMixer. CT‐phantom studies (**Figure** **1**A,B) indicated that GEM mixed with 20% Ta generated satisfactory visibility without any streak artifact. Fluoroscopy experiments showed that nano‐Ta could not be uniformly distributed throughout GEM causing poor visibility; thus, subsequent experiments used the micrometer‐size Ta particles in GEM (Figure 1C,D). While 50% iohexol‐GEM was the most visible on fluoroscopy, this formulation was not injectable through catheters. To test the visibility of Ta‐GEM in vivo, the hepatic vein in the pig liver was accessed percutaneously under ultrasound guidance using a 21 G needle; in real time, both US and fluoroscopic imaging were performed during Ta‐GEM injection indicating sufficient visibility (Figure 1E,F; white arrow indicates the same area in both images). US imaging showed that GEM is echogenic, allowing direct visualization; furthermore, shear‐wave US imaging indicated that the addition of Ta did not cause a significant increase in the stiffness of the embolic agent when compared to other samples with Ta (Figure 1G). Next, we performed standard T1 and T2‐based MRI of 20% Ta‐GEM loaded into a syringe. As expected, T1 sequences produced a dark image, and T2‐based sequences produced a bright image; this was expected given MR properties of Ta and the hydrogel nature of GEM (Figure 1H–K). Analysis of GEM including micro‐CT, CT, MRI, and scanning electron microscope (Figure 1L) imaging consistently demonstrated uniform dispersal of Ta in GEM representing effective speed mixing technique; this is important for providing a homogeneous signal during imaging. Thus, these results support that microsized Ta loading into GEM can be detected through X‐ray fluoroscopy, US, CT, and MRI and can provide a high‐quality homogeneous signal.

### 2.2 Injectability and Analysis of the Shear‐Thinning Property of Ta‐GEM

**Figure 2:**
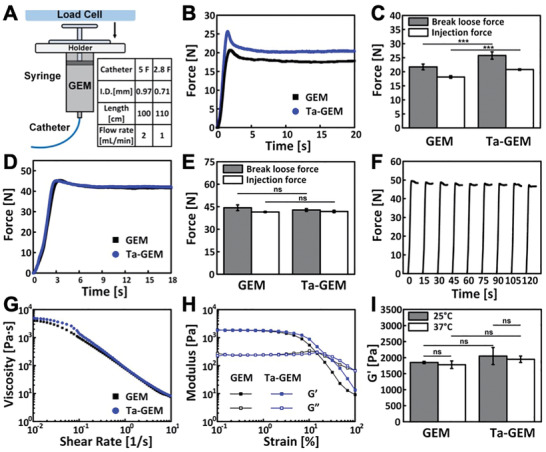
Physical and mechanical characterization of Ta‐GEM. A) Schematic of injection force testing. Table indicates the catheters and the parameters used during testing (I.D.: inner diameter of the catheter). B) Representative injection force curves of GEM and Ta‐GEM in a 1 cc syringe injected through 110 cm, 2.8F microcatheter at 1 mL min^−1^. C) Summary of break loose force and injection force of GEM and Ta‐GEM in a 2.8F microcatheter system (*n* = 5). D) Representative injection force curves of GEM and Ta‐GEM, respectively, loaded in a 3 cc syringe and injected through 100 cm, 5F catheter at 2 mL min^−1^. E) Summary of break loose force and injection force of GEM and Ta‐GEM in a 5F catheter system (*n* = 5). F) Injection force profile of an interrupted delivery (10 s delivery, followed by 5 s dwell time) of Ta‐GEM through a 5F catheter at 2 mL min^−1^. G) Flow curves comparing GEM and Ta‐GEM at 37 °C showing shear‐thinning properties. H) Amplitude sweeps of GEM and Ta‐GEM at 37 °C. I) Summary of storage modulus kinetics of GEM and Ta‐GEM at 25 and 37 °C (*n* = 3). Data are mean ± SD for (C), (E), and (I).

The capability to hand inject a syringe‐filled Ta‐GEM connected to a clinical catheter is a key practical requirement for the design of GEM. Previously, we have shown that changing the composition of gelatin and silicate nanoparticles in GEM can alter the mechanical, rheological, and bioactive properties of nanocomposite hydrogel.^[^ ^15^ ^]^ In particular, we showed that an increase in the gelatin ratio resulted in a decrease in swellability and an increase in degradability, while an increase in silicate nanoparticles resulted in a decrease in degradability and an increase in shear‐thinning properties. Based on these results, in this study, we applied the optimized Ta microparticles in Figure 1 to the GEM composition (gelatin:silicate nanoplatelets:ultrapure water 1.5:4.5:94) that showed the appropriate hemostatic properties and shear‐thinning effect in previous studies.^[^ ^12^ ^]^ To determine the effect of Ta addition to the GEM, the injectability of GEM and Ta‐GEM through 2.8 French (110 cm) and 5 French (100 cm) clinical catheters was examined using compression testing (**Figure** **2**A). In both systems, Ta‐GEM demonstrated comparable injectability to that of GEM, suggesting that the addition of Ta particles does increase the injection force, but the changes did not significantly impact injectability (2.8 French catheter, Figure 2B,C; 5 French catheter, Figure 2D,E). Next, we investigated the effect of interrupting delivery; no significant impact on the injection force was again observed (Figure 2F). The ability to perform intermittent delivery with real‐time evaluation of embolization is an important property; that is not possible with current technologies.

In addition to injectability, the influence of Ta microparticles on GEM's mechanical property was analyzed using rheological studies. The flow curves of GEM and Ta‐GEM demonstrate similar shear‐thinning characteristics (Figure 2G). To assess the embolic strength of GEM and Ta‐GEM, we performed amplitude oscillation tests to measure their storage modulus *G*′ and loss modulus *G*″. Figure 2H demonstrates that both GEM and Ta‐GEM are viscoelastic, and they possess *G*′ values that are ≈10 times of *G*″. Furthermore, no significant change in *G*′ (measured at 0.1% strain) was observed in Ta‐GEM compared to GEM (Figure 2I), suggesting that Ta had minimal impact on GEM's mechanical properties. These results suggested comparable embolic strength between GEM and Ta‐GEM.

### 2.3 Evaluation of Thrombogenicity, Stability, and Sterility of Ta‐GEM

**Figure 3:**
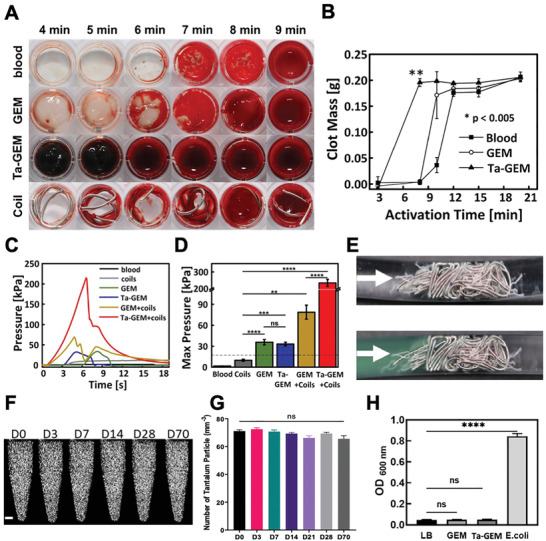
Evaluation of blood thrombogenicity and sterility of Ta‐GEM. A) Time to thrombosis of blood mixed with GEM, 20% w/w Ta‐GEM and commercially available fibered coil. B) Average mass of formed clot over time showing enhanced thrombogenicity with Ta‐GEM compared to blood alone or GEM alone. C) Resistance pressure curves in in vitro embolization tests. D) Graphic summary of the data in (C) showing peak pressure is maximally generated by Ta‐GEM + Coils compared to coil alone (**p < 0.01, ***p ≤ 0.001, ****p < 0.0001, data represent the mean ± SD, with *n* = 3). E) Photograph of fibered coils inside the vascular model with and without GEM (green). F,G) Ta‐GEM in tubes were kept in a 37 °C incubator up to 70 d. At D0, 3, 7, 14, 28, and 70, the tubes were imaged using a micro‐CT scanner and image analysis was performed; coronal views of the tubes are shown with the white bright spots representing the Ta. The number of Ta microparticles was counted in axial views at multiple levels and presented in (G). Statistical analysis was done using one‐way ANOVA, Tukey's multiple cooperation test (ns: not significant). H) Graphic summary of sterility test of GEM and Ta‐GEM mixed in LB broth and incubated for 7 d at 37 °C; LB broth inoculated with *E. coli* bacteria was used as a positive control. Optical density was measured in each aliquot at 600 nm. Results are mean ± SEM (**p < 0.01, ****p < 0.0001; *n* = 4).

Enhanced thrombogenicity of Ta‐GEM is a favorable trait in an embolic agent to achieve vascular occlusion. Blood coagulation in aliquots of citrated human blood mixed with Ta‐GEM was compared to standard coils used in patients. Both coils, GEM and Ta‐GEM demonstrated clot promoting properties when compared to blood alone; however, when the mass of the clot is measured, Ta‐GEM demonstrated a significant rise in mass beginning at 3 min compared to GEM and blood alone (**Figure** **3**A,B). These results suggest that Ta‐GEM has enhanced clot formation properties, which may further stabilize the embolic material at the blood–GEM interface.

To confirm the embolization ability of the Ta‐GEM, in vitro embolization experiments were performed (Figure 3C–E). The displacement pressures of GEM and Ta‐GEM are similar (35.8 ± 3.9 and 33.2 ± 2.6 kPa, respectively; *p* = 0.5), but significantly higher than coils alone (9.9 ± 1.5 kPa, *p* < 0.0001). More importantly, when Ta‐GEM is combined with coils, the displacement pressure reaches up to 234.5 ± 21.4 kPa or nearly 15 times greater than the standard physiologic systolic pressures in humans. The additional strength from GEM combined with coils may be due to increased friction between Ta particles and coils. We further evaluated the stability (Figure 3F,G) and sterility (Figure 3H) of Ta‐GEM; Ta‐GEM was incubated at 37 °C up to 70 d and micro‐CT analysis was performed to evaluate the uniformity of the Ta distribution and sterility over time. Ta‐GEM samples remarkably demonstrated consistent uniform distribution of the Ta throughout the study period. Following sterile preparation of the Ta‐GEM samples, they remained sterile despite storage in a 37 °C incubation chamber for 70 d (Figure 3H). Taken together, these results indicate that the addition of Ta microparticles does not lead to phase separation within GEM and remain sterile over time, and significantly increases GEM thrombogenicity allowing faster embolization and promotes significant synergistic effect when combined with conventional embolization techniques using coils.

### 2.4 Efficacy of Ta‐GEM Embolization of the Swine Iliac Artery: Nonsurvival Model

**Figure 4:**
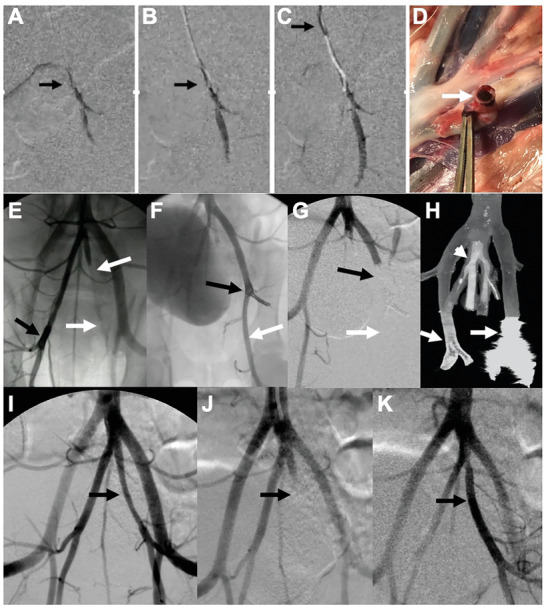
Assessing GEM embolization in the anticoagulated swine iliac artery. A–C). Serial digital subtraction angiography (DSA) images during Ta‐GEM injection into iliac artery is shown (for real‐time video, see Movie S1, Supporting Information). These images demonstrate instant occlusion upon exit from the catheter tip (arrow) without distal fragmentation. D) On necropsy, iliac artery containing Ta‐GEM is shown (arrow). E) DSA showing failure of coils to achieve occlusion (black arrow; contrast flows through the coil mass inside the artery). White arrows in the contralateral iliac artery show complete occlusion of the artery with GEM. F) Contrast injection in the iliac artery shows contrast flowing through the coil mass (black arrow) and beyond (white arrow). Following the delivery of Ta‐GEM to the coil mass in (F), DSA image in (G) shows complete occlusion (black arrow) with no break‐through blood flow (white arrow). H) Coronal micro‐CT image of (G) shows Ta‐GEM (arrowhead) uniformly casting the internal iliac arteries without artifact; in contrast, the coil mass (arrow) produced significant streak artifact limiting assessment of the artery and adjacent branch. I–K) Representative images of an internal iliac artery initially embolized with Ta‐GEM and later retrieved using the Penumbra aspiration catheter (black arrow). K) Restoration of flow following retrieval of GEM ≈3 h after embolization of the artery.

Based on the results of visualization, mechanical testing, and coagulation tests, we investigated the efficacy of Ta‐GEM embolization in the swine arterial system. Experiments were designed to evaluate the performance of Ta‐GEM compared to coils in the embolization of first‐order arteries in a state of extreme anticoagulation. Using clinical tools and imaging equipment, a combination of wire and a catheter were used to deliver the catheter tip to the distal abdominal aorta. Digital subtraction angiography (DSA) demonstrated the characteristic split of the aorta into the paired external and internal iliac arteries. Following injection of 20–30 000 units of IV heparin to achieve ACT >300 s, one of the iliac arteries was catheterized; ≈6–7 cm of the iliac artery was subsequently embolized with Ta‐GEM through a 5 French Bernstein catheter or a 2.8 French microcatheter (**Figure** **4**; real‐time delivery of Ta‐GEM in vivo and on necropsy is demonstrated in Figure 4A–D and Movies S1 and S2, Supporting Information). DSA from the aortic bifurcation following embolization demonstrated immediate and complete focal occlusion of the target artery. Next, detachable, 0.018 or 0.035‐in.‐thick coils of various lengths were used to embolize an iliac artery; despite deploying ≈50–80 cm of coils in a state of ACA >300, these coils did not achieve hemostasis for the duration of the experiment, which is consistent with clinical experience (Figure 4E,F; black arrow). As a comparison, the contralateral iliac artery was embolized with GEM leading to instant hemostasis (Figure 4E, white arrows). Time to embolization was also tracked since during emergency procedures rapid delivery of the embolic agent to achieve hemostasis is of paramount importance. On average, embolization time using Ta‐GEM was 40× faster than coil embolization (*p* < 0.00001); this demonstrates the simplicity and practicality of Ta‐GEM delivery using clinical catheters >100 cm in length, which is a unique property of this hydrogel.

We further investigated whether coils that failed to achieve hemostasis could be subsequently rescued by Ta‐GEM injection into the coil mass and whether Ta‐GEM could be retrieved from the artery following injection. To our knowledge, there are no embolization coils that can achieve hemostasis in a state of anticoagulation (ACT > 300) or coils that are designed to be removed after delivery. In eight cases where coils failed in a state of anticoagulation (i.e., Figure 4E,F), 2–3 cc of Ta‐GEM injection into the proximal end of the coil mass led to instant hemostasis and occlusion (Figure 4F,G, white arrow). Micro‐CT imaging of the embolized segments harvested following necropsy demonstrated uniform casting of the iliac arteries with Ta‐GEM without any CT imaging artifact even at high‐resolution imaging, allowing the ability to assess outcomes of the intervention (Figure 4H, arrowhead). However, in the adjacent arterial segment that was coil embolized, significant streak‐artifact was noted precluding the assessment of the embolized and adjacent arteries (Figure 4H, white arrow). These streak artifacts are a common occurrence in patients who receive CT scans; they limit the evaluation of the treatment zone and, in cerebral aneurysm cases, often leads to invasive angiography to assess for embolization efficacy.

Nontarget embolization is always a concern in embolization procedures. As a safety mechanism, we evaluated whether Ta‐GEM could be retrieved in the event of an unintended or accidental nontarget embolization. In five cases, the iliac artery was embolized using Ta‐GEM; after up to 3 h, Ta‐GEM was successfully retrieved in its entirety using the Penumbra Aspiration catheter system (Figure 4I–K), a common device used for stroke thrombectomy. These results indicate that Ta‐GEM could be retrieved; also suggests that, for the first time, temporary embolization may be a possibility where Ta‐GEM could be retrieved to restore normal flow, for example, in vascular trauma patients.

### 2.5 Efficacy of Ta‐GEM Embolization of the Swine Iliac Artery: Long‐Term Survival Model

**Figure 5:**
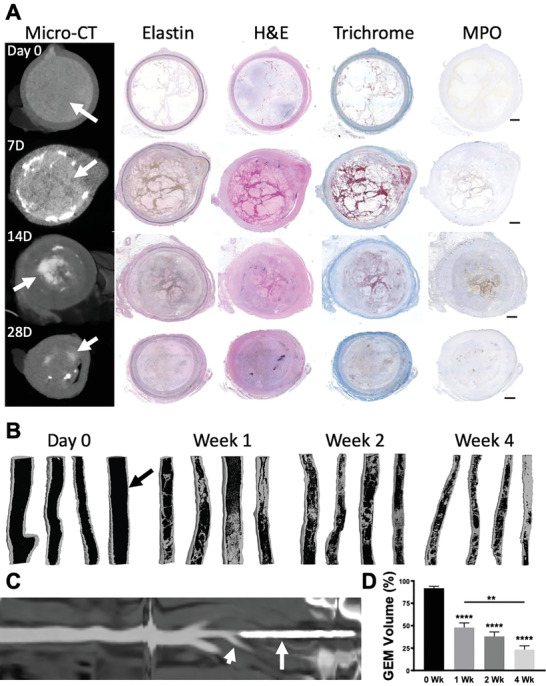
Assessing swine internal iliac artery occlusion by computed tomography and histology. A) A panel of micro‐CT images and their corresponding cross sections of the internal iliac artery on histology at 0, 7, 14, or 28 d following embolization were stained with H&E, VG Elastin, Masson's trichrome, and myeloperoxidase (MPO), respectively. At Day 0, there is uniform occlusion of the artery with GEM (arrow). Subsequently, a concentric fibroinflammatory reaction ensues that leads to biodegradation of GEM. B) 3D coronal rendering of reconstructed high‐resolution micro‐CT scans of the explanted pig internal iliac arteries following segmentation of GEM inside each vessel at each time point are shown (arrow points to GEM, black color inside artery; connective tissue is depicted as gray). Over time, there is progressive biodegradation of GEM and increase in fibrosis (gray). C) Reconstructed linear depiction of the aorta to the iliac artery showing Ta‐GEM inside the left internal iliac artery (arrow) and patent right internal iliac artery (arrowhead). D) Quantitation of Ta‐GEM over time from the micro‐CT segmentation dataset in (B) is shown as a graph revealing progressive decrease of Ta‐GEM inside the artery. Data are the mean ± SEM with *n* = 4, * *p* < 0.0001, ** *p* = 0.007 using ANOVA and Tukey's post hoc analysis.

We investigated the long‐term outcomes of using Ta‐GEM in the embolization of swine iliac arteries while receiving daily antiplatelet therapy for anticoagulation. Following up to 28 d of survival postembolization, the animals underwent contrast‐enhanced CT angiogram (CTA) and then were euthanized. All 16 animals demonstrated persistent embolization at each time point with no evidence for nontarget embolization on CTA imaging (**Figure** **5**). The embolized iliac arteries were occluded devoid of any flow to suggest recanalization; lower hindlimb perfusion from the aorta to the ankles via collaterals were also preserved (Figure S1A–C, Supporting Information). There were no focal bright spots within the pelvic or hindlimb muscle compartments to suggest fragmentation and dislodgement of Ta‐GEM (Figure S1B, Supporting Information). A board‐certified radiologist‘s review of the pig CTA images revealed unremarkable findings (Figure S1D–L, Supporting Information); for example, there was no evidence for lymphadenopathy, and the solid organs including the lungs, spleen, kidneys, and liver were normal. Subsequently, during necropsy, specimens from these organs were also collected for histologic evaluation; a review of these slides by a board‐certified pathologist showed normal findings. During necropsy, the pelvic arteries were also dissected and immediately imaged using a high‐resolution micro‐CT scanner. Image analysis of micro‐CT data compared to corresponding histology sections revealed progressive biodegradation of the Ta‐GEM over the four weeks (Figure 5). Segmented and 3D reconstructed iliac arteries using the micro‐CT dataset ranging from 700 Gb to 1 TB show progressive loss of Ta‐GEM volume (Figure 5B, the black inside each artery represents Ta‐GEM) and a decrease in the luminal diameter of the embolized arteries. After four weeks, micro‐CT volumetric analysis revealed that ≈75% of the Ta‐GEM had biodegraded and replaced by connective tissue as demonstrated by trichrome stain (Figure 5A,D). On Day 0, corresponding histology images showed uniform occlusion of the iliac artery with no significant tissue reaction and the absence of cellular infiltration (Figure 5A). However, over time, a concentric fibro‐inflammatory reaction ensues in the arterial lumen rich in macrophages, myofibroblasts, and fibrin resembling granulation tissue. The biomaterial becomes infiltrated with MPO‐positive granulocytes and scattered multinucleated giant cells as Ta‐GEM gradually gets replaced with connective tissue. The majority of the granulocytes were within the lumen of the artery (Figure 5A and Figure S2, Supporting Information) and both the internal and external elastic laminas showed evidence for disruption with breakdown of the elastic fibers of the intima and media layers and marked fibrosis of the media layer (Figure 5A and Figure S2C–E, Supporting Information). With progressive fibrosis of the lumen, the luminal diameter was decreased at four weeks following an initial distention with Ta‐GEM embolization (Figure 5, H&E and Trichrome stain). The thickness of the tunica media layer of the arterial wall did not change over four weeks.

To assess for systemic toxicity and inflammatory response to Ta‐GEM, complete blood count (CBC), basic metabolic panel (BMP), liver function tests (LFT), and a cytokine array analysis were performed; samples before embolization were compared to blood samples obtained just prior to sacrifice. Blood tests before and after embolization including CBC, LFTs, and BMP were unremarkable (*p* > 0.05, Table S1, Supporting Information); this is a significant result because it indicates that Ta‐GEM does not consume platelets, activate acute phase reactants, increase white blood cell counts because of an infection, or injure solid organs. Cytokine array analysis of the blood before and after embolization demonstrated no signs of inflammation, with most factors reduced in postembolization samples (Table S2, Supporting Information). Thus, these blood tests, cytokine analysis, histology, and cross‐sectional imaging of the pigs further demonstrate that Ta‐GEM is biocompatible and safe without evidence of an adverse systemic response.

### 2.6 Efficacy of Ta‐GEM Embolization of the Swine Renal Artery: Acute and Long Term Survival Model to Assess for Recanalization

Iliac artery embolization demonstrated that Ta‐GEM is stable with durable occlusion over 28 d. However, recanalization of the embolized arterial segment could not be excluded with certainty since CT images distal to the embolized iliac artery revealed contrast enhancement; this likely resulted from cross‐pelvic collateral flow bypassing the embolized segment. To assess for possible recanalization of Ta‐GEM over time, the main renal artery in 16 pigs was embolized. The kidney is comprised of end‐organ arteries; successful embolization of the main renal artery should result in distal ischemia and uniform atrophy of the kidney. However, any recanalization of the Ta‐GEM would lead to the restoration of blood flow and reduced or absence of atrophy. Furthermore, because the renal artery is an end‐artery, the smallest vessel that could be embolized with Ta‐GEM was also determined and confirmed by histology.

**Figure 6:**
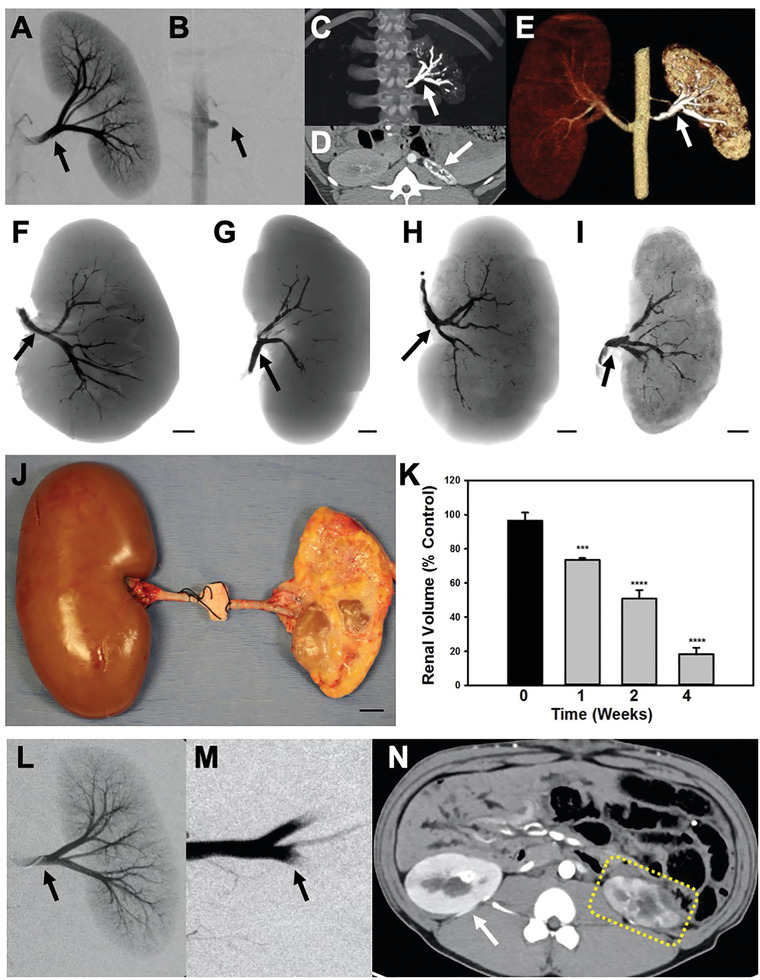
Embolization of the swine renal artery using Ta‐GEM and comparison to gelfoam. A,B) Fluoroscopic images before and after renal artery embolization with Ta‐GEM; following injection of 2–3 cc of Ta‐GEM, there is complete absence of renal arterial flow to the kidney (arrow). C) The maximum intensity projection image shows the left main renal artery and segmental branches occluded with Ta‐GEM (arrow). D) Axial CT and E) 3D reconstructed image shows the markedly atrophic kidney with Ta‐GEM in renal artery. F–I) Representative coronal images of the embolized kidneys with Ta‐GEM at 0, 1, 2, or 4 weeks, respectively. The cortex of the kidney is devoid of Ta‐GEM. J) Photograph showing the gross appearance of the embolized kidney at four weeks following necropsy with compensatory hypertrophy of the contralateral kidney. K) Summary of total kidney volume measurements following renal artery embolization (***p < 0.001, ****p < 0.0001 using one‐way analysis of variance, *n* = 4 in each group). L,M) DSA image before and after embolization of renal artery using gelfoam (black arrow). N) At two weeks postembolization, axial CT image shows enhancement of the embolized kidney and blood flow in the arteries despite successful renal artery embolization (dashed yellow outline). Overtime there is progressive atrophy of the kidney. Data reported as the mean ± SEM.

Using a 5 French Cobra catheter, the main renal artery was catheterized, which was confirmed by DSA imaging (**Figure** **6**A). Subsequently, 2–3 cc of Ta‐GEM was injected leading to the instant casting of the artery and occlusion (Figure 6B). DSA imaging from the aorta using the Cobra catheter consistently showed complete occlusion of the renal artery. Just prior to necropsy, imaging using a clinical CT scanner was performed (Figure 6C–E); these revealed the absence of flow to the embolized kidney and progressive atrophy over the 28 d study period. By four weeks, the embolized kidney demonstrated a consistent, ≈4‐fold decrease in the kidney volume without any flow in the embolized renal artery or any distal arterial segment (Figure 6F–K); these data, including extensive CT image analysis, suggested that there was no evidence for recanalization.

Next, we assessed for any evidence for fragmentation and nontarget embolization; because the kidney is an end‐organ artery, the 5–8 *μ*m capillaries would capture any dislodged Ta‐GEM and would be visible by micro‐CT, near‐infrared spectrum imaging, and by histology (Figure S3A–C, Supporting Information). This extensive analysis revealed that the cortex of the kidney did not contain any evidence for Ta‐GEM. On further analysis, to determine the smallest artery that Ta‐GEM could flow into, a 4 French Fogarty balloon was inflated in the main renal artery to fix its position followed by Ta‐GEM injection through this Fogarty catheter; injection was stopped when the Fogarty balloon demonstrated displacement. Subsequent high‐resolution micro‐CT imaging revealed that the smallest artery Ta‐GEM could be detected, measured ≈320 *μ*m (Figure S3D–F, Supporting Information). Furthermore, evaluation of the chest by a board‐certified radiologist using axial and reformatted coronal images revealed no evidence of focal hyperdensity or atelectasis to suggest migration of the embolic material from the renal arterioles to the capillaries. Axial, coronal, and sagittal images of the brain and extremity digits were also negative for nontarget embolization and any evidence for ischemic changes or microvascular infarcts.

Finally, the embolization performance of GEM was compared to gelfoam, which is clinically used today for hemorrhage control. Similar to the gelatin used in GEM, gelfoam is also comprised of porcine gelatin. Precut gelfoam (EmboCube, Meritt Medical) was mixed with 50% v/v saline and 50% iohexol to create a slurry. These were subsequently used to embolize the main renal artery to hemostasis (Figure 6L,M). Following two weeks of survival, animals were imaged using a clinical CT scanner; these images demonstrated contrast enhancement of the kidney resulting from significant recanalization and persistent blood flow within the renal artery (Figure 6N). This was confirmed by the absence of gelfoam inside the renal artery at necropsy and histology. Taken together, these data suggest that GEM outperforms coils and gelfoam, the current clinical tools used today for embolization.

## 3 Discussion

Transcatheter embolization has witnessed tremendous growth over the past several decades. For example, treatment of most cerebral aneurysms, nontraumatic and some traumatic hemorrhage, and gastrointestinal bleeding have shifted from open surgical management to coil embolization. Here, we show a novel composition for GEM comprised of gelatin, nanosilicates, and microparticles of Ta with shear‐thinning properties that allow delivery using standard clinical catheters. Ta‐GEM has thrombotic properties similar to coils, can be sterilized, and can be visualized under ultrasound, CT, MRI, and X‐ray fluoroscopy. It has a displacement pressure many‐fold greater than systolic pressure, and when combined with coils, the displacement pressure exceeds 1600 mmHg. In pigs, Ta‐GEM can be visualized in real time during delivery into first‐order arteries achieving instant embolization using clinical catheters that is 40× faster to deploy than coils and Ta‐GEM remains in place without fragmentation or migration. Arterial occlusion with Ta‐GEM is durable without any evidence for recanalization; biodegradation of Ta‐GEM in pigs is rapid with only 25% remaining at four weeks. In pig kidneys, the smallest artery that can receive Ta‐GEM is ≈320 *μ*m implying that Ta‐GEM will not reach venous circulation. In addition, Ta‐GEM is safe without evidence for toxicity on standard laboratory tests, cytokine array analysis, and CT imaging.

There are several limitations to this study. The survival experiments in the swine model of embolization lasted 28 d; analysis of imaging and tissue sections at 28 d indicated that most of the Ta‐GEM had biodegraded. However, the time point when Ta‐GEM is undetectable by imaging and histology was not determined; this likely will occur between the 2 and 3 months postembolization time point. We also did not evaluate the mode of excretion of the byproducts of NC breakdown and whether any accumulation in organs occurred. It has been reported that NC use is safe,^[^ ^20^ ^]^ and its byproducts are likely excreted via the hepatic system.^[^ ^21^ ^]^ In addition, MRI imaging prior to the sacrifice of the pig models could not be performed due to limitations in access to the scanner. Finally, while Ta‐GEM appears to be visible on sonography, we did not perform US‐guided embolization of arteries since we focused on catheter‐directed endovascular embolization in this study.

A bioengineered material that can be safely used in vivo to achieve rapid arterial embolization under real‐time visualization regardless of coagulopathy represents a major advance in the field of materials research and vascular embolization. As medicine transitions to approaches that are more minimally invasive, easy, safe, and efficacious delivery of novel embolics to diseased tissue will be critical to achieving healing, tumor shrinkage, and hemorrhage control. While the traditional approaches to embolization were to achieve hemostasis only, we believe the next generation of embolics will comprise of biomaterials that can not only achieve instant hemostasis but also provide therapeutics, real‐time imaging capability, and flexibility with respect to the mode of delivery and retrievability. In summary, Ta‐GEM has numerous advantages over coils and gelfoam used today and would be a promising embolic agent for the treatment of vascular embolization.

## 4 Experimental Section

##### GEM Synthesis and Sterilization

Iohexol‐free GEM was prepared by mixing 18% (w/v) gelatin (Type A, Sigma Aldrich, St. Louis, MO), 9% (w/v) silicate nanoplatelets (Laponite XLG, BYK USA Inc., Rochester Hills, MI), and ultrapure water at a weight ratio of 1:6:5, according to a previously developed protocol.^[^ ^12^, ^13^ ^]^ To introduce radiopacity, GEM was mixed with iohexol or tantalum particles. Iohexol solution (Omnipaque 350 mgI mL^−1^, GE HealthCare, MA) was mixed into GEM to achieve a 10% w/w final concentration. Ta microparticles with an average size of 2 µm (Alfa Aesar, Haverhill, MA) or Ta nanoparticles <25 nm particle size (Sigma‐Aldrich, St. Louis, MO) were mixed with GEM at various w/w levels to form Ta‐GEM hydrogel (e.g., 20% w/w Ta GEM). The homogeneous mixing of all GEM formulations was achieved by using a SpeedMixer (FlackTek Inc., Landrum, SC). To sterilize Ta‐GEM, RS2000 irradiator system (RAD.SOURCE) was used to expose Ta‐GEM loaded syringes to 160 kV, 25 mA of ionizing irradiation dose equivalent to 11 cGy min^−1^ for a total of 12 000 rads based on an established protocol to eradicate bacteria and fungus.^[^ ^22^ ^]^ To confirm sterility, standard microbial growth assay using LB agar plates or LB broth with and without 100 mg mL^−1^ ampicillin was performed. LB broth tubes containing 10^7^–10^8^ CFU of chemically competent *E. coli* bacteria were used as a positive control. LB broth tubes which had 0.1 mL PBS alone served as a negative control. Quadruplicate tubes from Ta‐GEM batches and each control were prepared and incubated on a shaking platform inside a 37 °C incubator for up to 7 d. The analysis was performed as previously described.^[^ ^12^ ^]^

##### Rheological Testing

Flow curves and amplitude sweeps were performed according to a previously developed protocol.^[^ ^12^ ^]^ The rheological evaluation of Ta‐GEM was performed using an Anton Paar MCR 302 rheometer (Anton Paar USA Inc., Torrance, CA). A sandblasted 25 mm diameter aluminum upper plate and an aluminum lower plate, with a 500 µm gap in between, were used for all measurements. Flow curves and amplitude sweeps (at 10 rad s^−1^) were obtained at 25 and 37 °C. For tests at 37 °C, the solvent trap was used, and the edge of the solvent trap was filled with water to provide a humidified environment. Data were acquired at least in triplicates for each experiment.

##### Analysis of Ta‐GEM Injectability

Injectability was examined using a mechanical tester equipped with a 100 N load cell (Instron, Norwood, MA) according to the previously established protocols.^[^ ^12^ ^]^ Briefly, Ta‐GEM was loaded into 1 or 3 cc Luer‐lock syringes (Medallion, Merit Medical, South Jordan, UT) and injected through a 110 cm 2.8 French ProGreat catheter (Terumo Medical Corporation, Somerset, NJ) or a 100 cm 5F Bernstein catheter (Cook Medical Inc., Bloomington, IN), respectively. Ta‐GEM containing syringes were inserted inside of a custom‐designed 3D printed holder, and the plunger was placed against the load cell plate; the compression force was applied at either 2 or 1 mL min^−1^ rate for 5F and 2.8F, respectively. To simulate injection during clinical practice, interrupted compression force was also applied for 10 s, followed by 5 s pause; this cycle was repeated eight times through a 5 French catheter. The generated break loose and injection forces overtime was acquired and plotted using Bluehill version‐3 software (Instron, Norwood, MA). Injectability testing was repeated at least five times for each condition.

##### Assessing the Effect of Ta‐GEM on Blood Clotting Time

Clotting time and thrombus weight were quantified using a previously developed protocol.^[^ ^12^, ^13^ ^]^ Briefly, 0.5 g of Ta‐GEM aliquots were weighed into 2 mL microtubes. Ta‐GEMs were centrifuged at 1000 RPM to standardize the blood interaction surface. Uncoagulated citrated blood was reactivated by adding 10% (v/v) 0.1 m CaCl_2_. 100 µL of activated blood was added to each Ta‐GEM sample and allowed to react for 3, 8, 10, 12, 15, or 20 min. At each time point, clotting was stopped by the addition of 200 µL of 0.109 m sodium citrate solution. Residual liquid was removed, isolating the clotted blood. The clotted blood was weighed in each test tube to determine the mass.

##### Percutaneous Endovascular Embolization in Pigs

This study was approved by the Institutional Animal Care And Use Committee (IACUC). Yorkshire pigs (S&S Farms, Brentwoods, CA) weighing 48 to 55 kg were acclimatized for at least 4 d. Anesthesia was induced using intramuscular injection of 5 mg kg^−1^ tiletamine‐zolazepam (Telazol, Zoetis), 2 mg mL^−1^ xylazine, and 0.02 mg kg^−1^ glycopyrrolate. Pigs were then placed in a supine position and intubated on an X‐ray compatible operating table (Pannomed Aeron, DRE, KY). Following intubation, anesthesia was maintained with inhalation of 1.5–3% Isoflurane. During the procedure, electrocardiogram, transcutaneous oxyhemoglobin saturation (*S*_pO2_), end‐tidal CO_2_ concentration, inspired oxygen fraction, and core temperature were monitored. Percutaneous access to the carotid artery was obtained using ultrasound guidance (ACUSON S2000, Siemens) and fluoroscopy (OEC9800 plus C‐Arm and OEC Elite CFD, GE Healthcare Systems, Chicago, IL). Access needle and wire were exchanged for a 5 French Bernstein catheter (Cook Medical). Over a GT‐glidewire (Terumo Medical), the tip of the catheter was advanced to the renal or the iliac artery using contrast‐enhanced fluoroscopy (350 mgI mL^−1^ Omnipaque, GE HealthCare, MA). Pigs received 10 000–30 000 IU of heparin IV, and ACT levels were documented using iSTAT analyzer (Abbot Laboratories Princeton, NJ). Ta‐GEM or EmboCube (Merit Medical) was delivered to the first‐order arterial branches iliac or renal artery using a catheter under real‐time fluoroscopic guidance. Syringes with the embolic agent were connected directly to the catheter using the Luer‐lock. In a subset, iliac arteries received metallic coils, including those from Medtronic, Terumo, Cook Medical, and Boston Scientific. The time to deployment and embolization of Ta‐GEM or coils was recorded for each procedure. Following embolization, angiography was repeated multiple times to assess vessel patency. Pigs were either euthanized 1 h postembolization (nonsurvival group) or at 1, 2, or 4 weeks postembolization (survival group). Prior to euthanasia, blood samples were obtained for analysis, and whole animal CTA imaging was performed. At necropsy, vascular tissues containing Ta‐GEM or coils were explanted for μCT imaging and histopathology evaluation. Tissues from the liver, spleen, heart, and normal kidneys were also obtained for histology review to assess for potential toxicity.

##### CT, US, MRI, and Fluoroscopy Acquisition Methods

CT acquisition was performed on a dual‐source scanner (Siemens Force, Siemens Healthineers, Erlangen, Germany). The spiral scan was performed at 150 and 80 kVp energy level, respectively, with a 0.6 mm detector size configuration. The MRI acquisition was performed on a 3.0 T scanner (Siemens Skyra, Erlangen, Germany). The 20‐channel head coil with the main body transmit coil was used to acquire the data. MRI acquisition consisted of axial single‐shot T2, axial dual‐echo in‐phase and out‐of‐phase, T1‐weighted imaging before, and balanced steady‐state free precession. The fluoroscopy acquisition was performed on a mobile C‐arm (OEC 9800 Plus, GE Healthcare, Illinois, USA).

##### Micro‐CT Imaging and Structural Analysis

Explanted embolized vessels were fixed with 10% formalin and incubated in 70% ethanol for 24 hours. Micro‐CT imaging was performed using SkyScan 1276 (Bruker, Kontich, Belgium) at 33 kV and 223 µA for rat vessels and 45 kV and 200 µA for pig arteries at a pixel size of 10 µm. Data analysis was performed using NRecon, CTvox, Data Viewer, and CTAn software (Bruker, Kontich, Belgium). To document structural changes in the injected Ta‐GEM, a quantitative morphometric analysis was performed to calculate surface‐to‐volume ratio, surface convexity index, fractal dimension, and structure model index as previously described.^[^ ^23^ ^]^ To calculate Ta‐GEM volume inside the embolized pig vessels, Mimics software (Materialise, Leuven, Belgium) was used to segment Ta‐GEM and the vessel lumen.

##### Histopathology and Immunohistochemistry

Paraffin‐embedded sections were stained with H&E, Masson's trichrome, or EVG elastin stain and immunostaining for myeloperoxidase (MPO; ab208670, Abcam) or CD68 (ab125212, Abcam) were performed, as previously described.^[^ ^12^ ^]^ Morphometric analysis was performed using Image analysis software (Celleste 4.1, Thermo Fisher Scientific). A blinded observer counted MPO or CD68 positive cells.

##### Multiplex Analyses of Serum

Selected biomarkers, including cytokines, chemokines, and growth factors were measured in serum samples obtained from aliquots of blood samples from rats at 0, 3, 7, and 21 d and from pigs at 0, 1, 2, and 4 weeks postembolization. The rat's serum samples were analyzed using the cytokine/chemokine array 27‐plex, while the pig samples were analyzed using the porcine cytokine/chemokine array 13‐plex (Eve Technologies, Calgary, CA). The analyte concentrations are expressed in picogram per mL.

##### Statistical Analysis

Analyses between groups at different time points were performed using analysis of variance (ANOVA), with post hoc multiple comparison procedures. A comparison between the two groups was calculated using the Student's *t*‐test. Statistical significance in intergroup differences was assessed at the 95% confidence level (*p* < 0.05). Data are presented as mean ± standard errors of the mean (SEM). All comparisons were obtained using GraphPad Prism 7 (GraphPad Software, Inc., La Jolla, CA).

## Conflict of Interest

R.O. and A.K. are the founders of a start‐up, Obsidio, Inc., which is based on shear‐thinning embolic materials.

## Supporting information

Supporting Information

Supplemental Movie 1

Supplemental Movie 2
