# Supplementary material for: Nanocomposite Hydrogel with Tantalum Microparticles for Rapid Endovascular Hemostasis
Source: Adv Sci (Weinh). 2020 Nov 30;8(1):2003327. doi: 10.1002/advs.202003327 (PMC7788497; doi:10.1002/advs.202003327)
Supplement: Supplementary file 1 — Supporting Information [file ADVS-8-2003327-s001.pdf]

WILEY-VCH

© 2020 Wiley-VCH GmbH

ADVANCED SCIENCE
Open Access

Supporting Information

for *Adv. Sci.,* DOI: 10.1002/advs.202003327

Nanocomposite Hydrogel with Tantalum Microparticles for Rapid Endovascular Hemostasis

Hasan Albadawi, Izzet Altun, Jingjie Hu, Zefu Zhang, Anshuman Panda, Han-Jjun Kim, Ali Khademhosseini, Rahmi Oklu**

Supporting Information123

Nanocomposite Hydrogel with Tantalum Microparticles for Rapid Endovascular Hemostasis

Hasan Albadawi, Izzet Altun, Jingjie Hu, Zefu Zhang, Anshuman Panda, Hanjun Kim, Ali Khademhosseini, Rahmi Oklu**

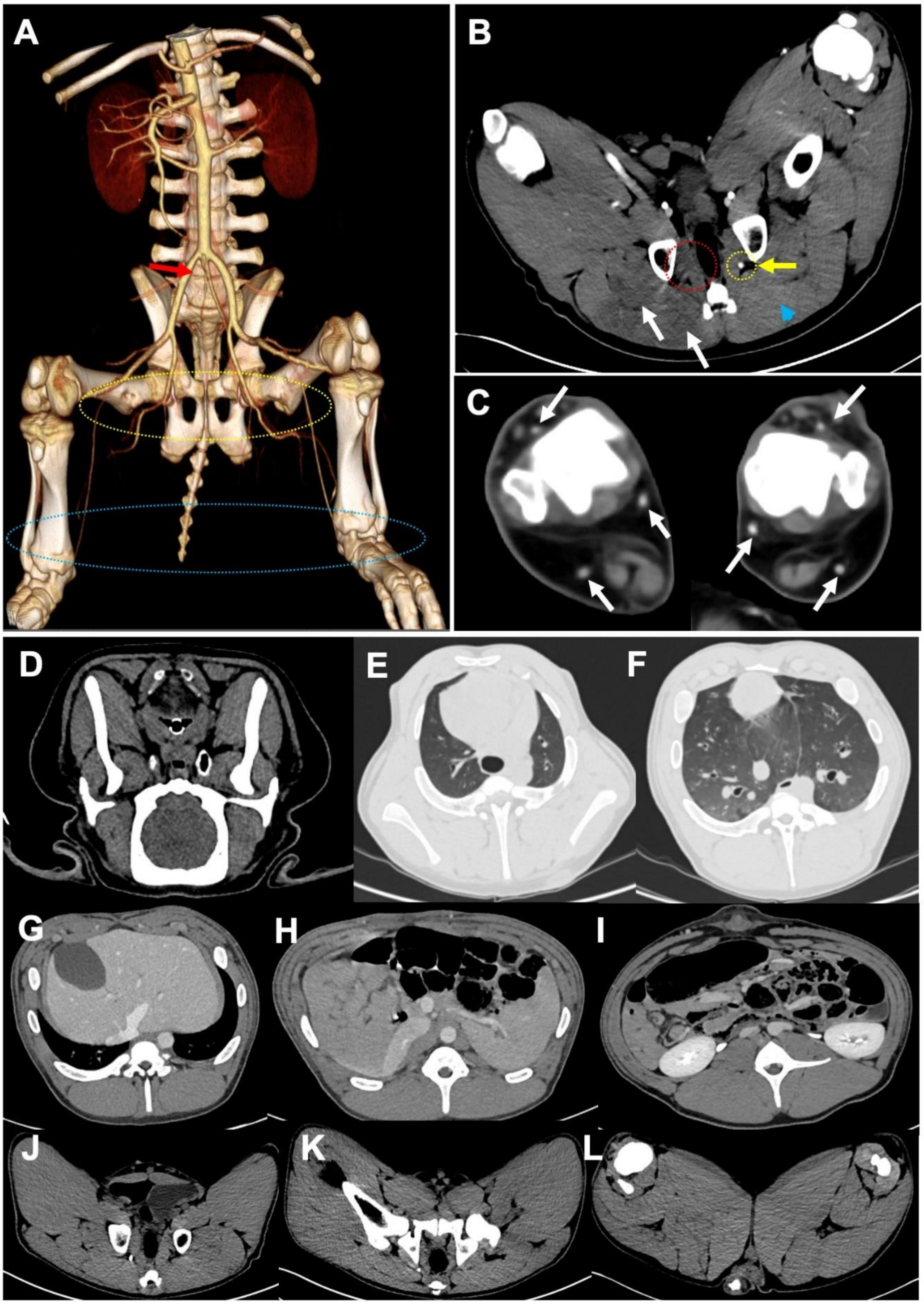

Figure S1. Assessing arterial occlusion in the pig and organ integrity with computed tomography angiography at 4 weeks post-embolization. **(**A) Reconstructed 3D image from a

contrast enhanced CT angiography shows absence of the right internal iliac artery (red arrow); this artery does not opacify with IV contrast because it is occluded with Ta-GEM. Axial image of the dotted yellow circle in (A) is displayed in (B). (B) Red circle shows absence of flow in the embolized artery with geographic hypodensity in the gluteal muscles indicating evidence of ischemia (white arrow). Contralateral artery (small yellow circle) is widely patent with no evidence of hypodensity or ischemia in the respective gluteal muscles (blue arrowhead). Axial image of the dotted blue circle in (A) is displayed in (C). (C) Axial image of the distal hindlimbs demonstrates normal run-off vessels (arrows) to the feet suggesting that there is no evidence for non-target embolization. Panel of axial CT images of the brain (D), heart (E), lungs (F), liver (G), spleen (H), kidneys (I), pelvis (J, K), hind limbs (L), reveal normal radiographic appearance.

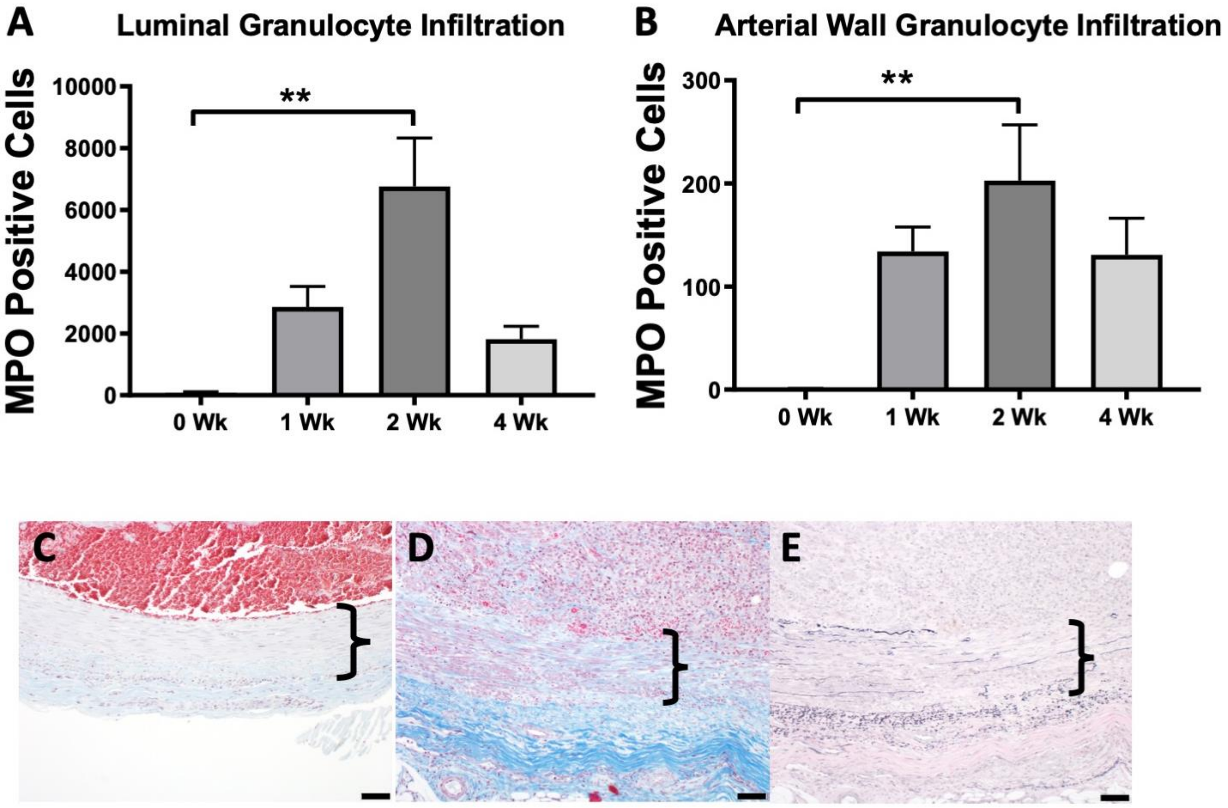

Figure S2. Quantitative analysis of inflammatory cell infiltration following internal iliac artery embolization and histology. (A) Summary graph of the average number of MPO expressing cells inside the lumen of the pig's internal iliac artery embolized with Ta-GEM; there is significant infiltration of MPO expressing cells at 2 weeks following embolization (** $p = 0.004$ using ANOVA with Kruskal-Wallis post hoc test. (B) Graph of MPO expressing cells infiltrating the arterial wall compartment show a marked increase at 2 weeks following embolization. ** $p = 0.007$ using ANOVA with Tukey's post hoc tests. Data are the mean ± SEM, n=4 for each data point. **(**C) On histology, at day 0 following embolization, Trichrome stain demonstrates an intact media layer. (D) At day 14, however, there is disruption and fibrosis of the media layer; (E) Elastin stain of the media layer shows disruption of the intima and media layers. Scale bar 50 microns.

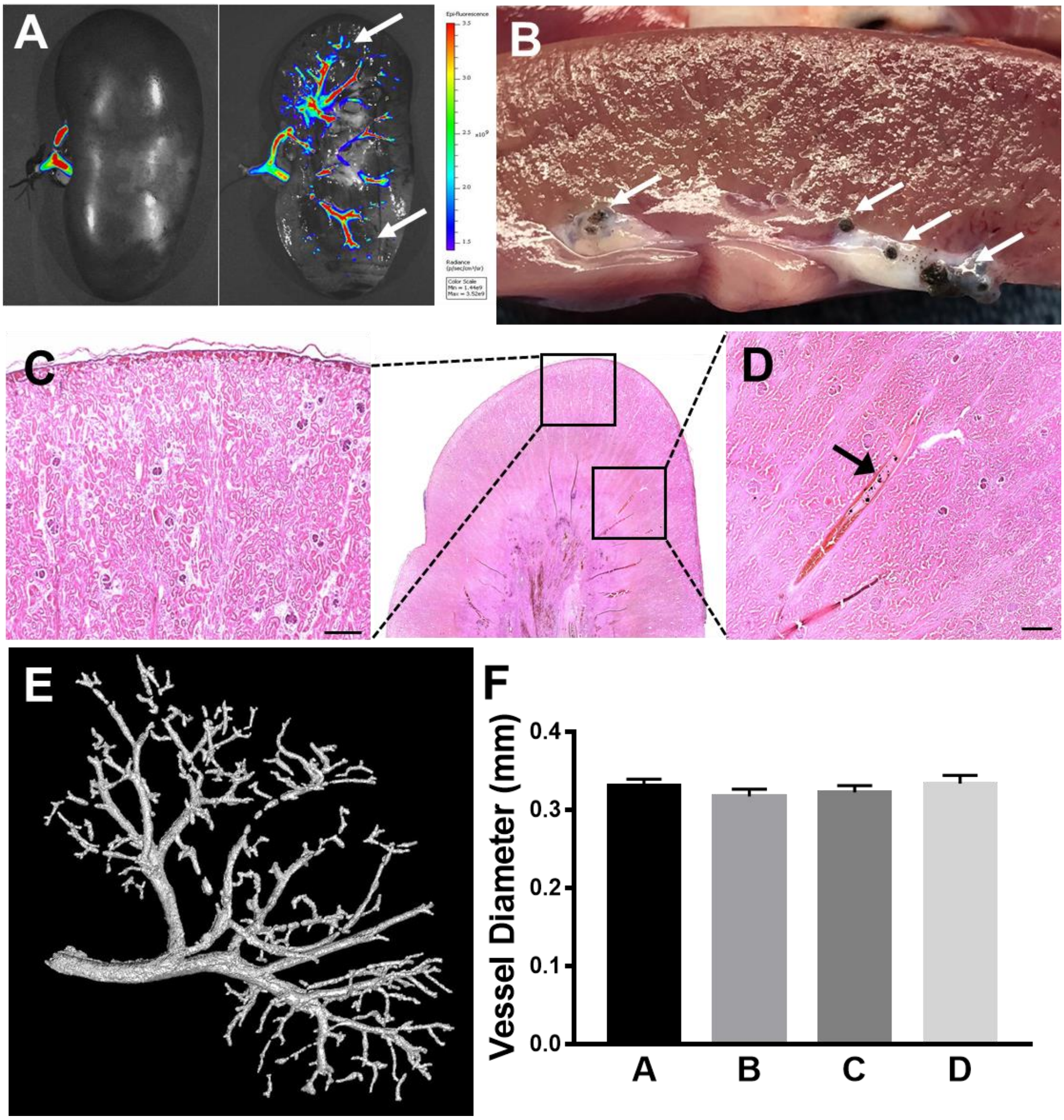

Figure S7. Assessing intravascular distribution of Ta-GEM following renal artery embolization in pigs using a Fogarty catheter. **(**A). A 4 French Fogarty catheter in the mid-main renal artery was inflated and Ta-GEM was injected continuously until the balloon demonstrated displacement. Following coronal transection of the kidney at necropsy, near-infrared imaging of this kidney embolized with Ta-GEM containing indocyanine green showed distribution of GEM in the main renal artery and in segmental arteries sparing the cortex. (B) Transverse section across the lower pole of the kidney in (A) shows arterioles embolized with Ta-GEM (arrows). (C) H&E of a slide showing the cortex demonstrates

normal glomeruli with absence of Ta-GEM in the cortex. (D) H&E image showing interlobar arterioles embolized with Ta-GEM. (arrow; scale; 500 µm). (E) The whole kidney in (A) was imaged ex vivo in a microCT scanner; these images show embolic material in blood vessels reaching proximal arcuate arterioles. (F) Graphic summary of arterial diameters measured in four kidneys reveal a mean vessel diameter of the smallest arteries embolized to be in the range of 320 µm.

	0 Week	1 Week	2 Week	4 Week
**WBC**	11.18 ± 1	15.5 ± 0.76	13 ± 1.67	24 ± 7
**Lymphocyte**	6.5 ± 0.5	9 ± 0.46	7.6 ± 0.58	15.5 ± 5.8
**Monocyte**	0.78 ± 0.1	1.15 ± 0.1	1 ± 0.08	1.97 ± 0.7
**Granulocyte**	4 ± 0.5	5.2 ± 0.3	4.5 ± 1	7.4 ± 1.3
**Lym%**	56 ± 6.9	59 ± 0.9	61 ± 3	59.3 ± 5.7
**Mon%**	6.0 ± 0.7	7 ± 0.1	6 ± 0.3	7.6 ± 0.4
**Gra%**	38 ± 6.3	34 ± 0.8	33 ± 3	33.1 ± 6.0
**HCT**	22.8 ± 0.6	26 ± 1.1	25 ± 2.2	27 ± 1.8
**MCV**	43.6 ± 1.1	45 ± 0.3	43 ± 1	44.8 ± 0.9
**RDWa**	26.7 ± 0.6	28 ± 0.4	25 ± 1	26.8 ± 0.5
**RDW%**	22 ± 0.9	22 ± 0.7	21 ± 1	21.5 ± 1.1
**HGB**	9 ± 0.2	10 ± 0.4	9.4 ± 0.8	9.8 ± 0.6
**MCHC**	38.4 ± 0.3	38 ± 0.3	3 9 ± 1	36.4 ± 0.2
**MCH**	16.8 ± 0.5	17 ± 0.2	17 ± 1	16.3 ± 0.3
**RBC**	5.2 ± 0.2	5.86 ± 0.3	5.82 ± 0.6	6.0 ± 0.3
**Platelets**	330 ± 57	371 ± 23	283 ± 79	319.0 ± 75
**MPV**	6.5 ± 0.2	6 ± 0.2	6 ± 0.1	6.5 ± 0.2
**TP g/dl**	5 ± 0.1	*6.5 ± 0.1	6.4 ± 0.14	6.7 ± 0.2
**ALP U/L**	186 ± 7	165 ± 16	156 ± 23	146 ± 9.0
**GLU mg/dl**	81±10.5	124 ± 6.6	112 ± 3.1	103 ± 7.0
**ALT U/L**	36 ± 1.7	*89.5 ± 21	42 ± 1.2	40.3 ± 3
**CRE mg/dl**	1.34 ± 0.06	1.5 ± 0.2	1.67 ± 0.1	*1.7 ± 0.2
**BUN mg/dl**	9 ± 0.9	14 ± 1.6	15.5 ± 1.22	21.8 ± 0.8

Table S1. Complete blood count and serum chemistry profile in pigs following arterial embolization with GEM. Table shows blood values measured at 0, 1, 2, or 4 weeks following internal iliac artery embolization. WBC, white blood cells; HTC, hematocrit; MCHC, mean corpuscular hemoglobin concentration; MCV, mean corpuscular volume; RDW, erythrocyte

distribution width; HGB, hemoglobin; RBC, red blood cells; MPV, mean platelet volume: ALP, alkaline phosphatase, CRE, creatinine; ALT, alanine aminotransferase; BUN, blood urea nitrogen. All measured values remained within the normal limits for Yorkshire swine. Results are mean ± SEM, unit, picogram per mL, n=4 in each group (*$p < 0.05$).

	0 Week	1 Week	2 Week	4 Week
**GM-CSF**	87 ±69	24±24	137±80	73±68
**IFNy**	14193±4335	2633±1244	9258±7924	7637±2928
**IL-1α**	57±19	12±5	60±41	26±12
**IL-1β**	537±186	130±60	597±392	205±116
**IL-1rα**	*2390±956	130±60	713±217	216±44
**IL-2**	445±161	134±54	502±335	262±128
**IL-4**	2964±1308	326±168	4206±3433	2140±1032
**IL-6**	106±33	36±22	212±169	89±61
**IL-8**	*355±118	79±26	298±166	88±26
**IL-10**	1311±475	219±53	1411±1065	668±394
**Il-12**	932±106	888±56	1355±68	1406±369
**IL-18**	1678±546	609±119	2200±1326	911±435
**TNFα**	468±327	35±30	27±17	30±18

Table S2. Serum levels of cytokines and chemokines following arterial embolization in pigs. Multiplex analysis of cytokines and chemokines at 0, 1, 2, or 4 weeks following internal iliac artery embolization. Results show no significant increase in all measured values. Results are mean ± SEM, unit, picogram per mL, n=4 in each group. GM-CSF, Granulocyte-macrophage colony-stimulating factor; IFNγ, interferon-gamma; IL, interleukin; TNF, tumor necrosis factor.
